# Long-term non-progression and risk factors for disease progression among children living with HIV in Botswana and Uganda: A retrospective cohort study

**DOI:** 10.1016/j.ijid.2023.11.030

**Published:** 2023-11-28

**Authors:** Samuel Kyobe, Grace Kisitu, Savannah Mwesigwa, John Farirai, Eric Katagirya, Gaone Retshabile, Lesedi Williams, Angela Mirembe, Lesego Ketumile, Misaki Wayengera, John Mukisa, Gaseene Sebetso, Thabo Diphoko, Marion Amujal, Edgar Kigozi, Fred Katabazi, Ronald Oceng, Busisiwe Mlotshwa, Koketso Morapedi, Betty Nsangi, Edward Wampande, Masego Tsimako, Chester Brown, Ishmael Kasvosve, Moses Joloba, Gabriel Anabwani, Sununguko Mpoloka, Graeme Mardon, Adeodata Kekitiinwa, Neil A. Hanchard, Jacqueline Kyosiimire–Lugemwa, Mogomotsi Matshaba, Dithan Kiragga

**Affiliations:** 1 Department of Medical Microbiology, College of Health Sciences, Makerere University, Kampala, Uganda; 2 Baylor College of Medicine Children’s Foundation Uganda, Kampala, Uganda; 3 Botswana-Baylor Children’s Clinical Centre of Excellence, Gaborone, Botswana; 4 Department of Immunology and Molecular Biology, College of Health Sciences, Makerere University, Kampala, Uganda; 5 Department of Biological Sciences, University of Botswana, Gaborone, Botswana; 6 Australian Medical Research, Hurstville, Australia; 7 University of Tennessee Health Science Center, Memphis, USA; 8 School of Allied Health Professionals, Faculty of Health Sciences, University of Botswana, Gaborone, Botswana; 9 Department of Molecular and Human Genetics, Baylor College of Medicine, Houston, USA; 10 Department of Pathology and Immunology, Baylor College of Medicine, Houston, USA; 11 Pediatric Retrovirology, Department of Pediatrics, Baylor College of Medicine, Houston, USA; 12 USDA/ARS/Children’s Nutrition Research Center, Baylor College of Medicine, Houston, USA; 13 Childhood Complex Disease Genomics Section, Center for Precision Health Research, National Human Genome Research Institute, National Institutes of Health, Bethesda, USA; 14 Uganda Virus Research Institute, Entebbe, Uganda

**Keywords:** LTNP, HIV progression, Pediatric, Africa

## Abstract

**Objectives:**

We utilize a large retrospective study cohort derived from electronic medical records to estimate the prevalence of long-term non-progression (LTNP) and determine the factors associated with progression among children infected with HIV in Botswana and Uganda.

**Methods:**

Electronic medical records from large tertiary HIV clinical centers in Botswana and Uganda were queried to identify LTNP children 0–18 years enrolled between June 2003 and May 2014 and extract demographic and nutritional parameters. Multivariate subdistribution hazard analyses were used to examine demographic factors and nutritional status in progression in the pre-antiretroviral therapy era.

**Results:**

Between the two countries, 14,246 antiretroviral therapy-naïve children infected with HIV were enrolled into clinical care. The overall proportion of LTNP was 6.3% (9.5% in Botswana vs 5.9% in Uganda). The median progression-free survival for the cohort was 6.3 years, although this was lower in Botswana than in Uganda (6.6 vs 8.8 years; *P* <0.001). At baseline, the adjusted subdistribution hazard ratio (aHR_sd_) of progression was increased among underweight children (aHR_sd_ 1.42; 95% confidence interval [CI]: 1.32–1.53), enrolled after 2010 (aHR_sd_ 1.32; 95% CI 1.22–1.42), and those from Botswana (aHR_sd_ 2; 95% CI 1.91–2.10).

**Conclusions:**

In our study, the prevalence of pediatric LTNP was lower than that observed among adult populations, but progression-free survival was higher than expected. Underweight, year of enrollment into care, and country of origin are independent predictors of progression among children.

## Introduction

Africa remains the global epicenter of the HIV epidemic; more than 70% of all people living with HIV/AIDS are in Africa. In 2021, UNAIDS estimates indicated that 150,000 new HIV infections and 99,000 deaths occurred among African children [1]. Before the initiative of universal antiretroviral therapy (ART), it was widely noted that some children would remain AIDS-free for more than 10 years and could maintain normal-for-age clusters of differentiation (CD4^+^) T-cell counts [2] – so-called long-term nonprogressors (LTNP). Children capable of controlling HIV infection present the opportunity for unique insights into the natural host immune responses, which could be essential for the development of novel therapeutics and vaccines [3]. LTNP has been noted among adults, particularly from Western populations; however, most studied individuals were infected via horizontal transmission, necessitating an estimated date of seroconversion. Unlike in adults, vertical HIV infection in pediatric cohorts provides a distinct advantage in estimating progression-free survival. The majority of infections occur (or are detectable) within 45 days of birth, with the remaining instances typically linked to breastfeeding or maternal seroconversion during breastfeeding [2,4,5]. Given the temporally fewer yet measurable childhood exposures [6], pediatric cohorts offer a valuable opportunity to accurately determine the proportion of LTNP. Further, the rapidly developing immunological system in children, forged through new exposures to infectious agents, is another crucial difference in pediatric HIV progression. For instance, children infected with HIV produce stronger de novo autologous HIV variant-specific CD8^+^ T-cell lymphocyte (CTL) responses than adults [7,8], and LTNP children are known to have more neutralizing antibodies and immune quiescence than their adult counterparts [9]. The factors associated with progression include host (genetics, sex, immunological profiles, cognitive factors) [8,10], pathogen (clade) [11], and environmental factors (psychosocial, diet) [10,12], which vary widely between populations. Understanding the demographic, clinical, and laboratory factors that contribute to HIV disease progression in general among children, could help to facilitate the prioritization of resources, especially in low- and middle-income countries.

As is the norm in adult literature, few studies on the prevalence of LTNP in children have been focused on Western populations [13–16]. A thorough examination of that literature, however, uncovers substantial clinical heterogeneity and small sample sizes among contributing studies, which limit the generalizability and applicability of the results. Nielsen et al., for example, estimated a 27% rate of LTNP among 143 children vertically infected with HIV. In contrast, Martin and colleagues observed a substantially lower estimate of 2.1% among 422 newborns in the USA classified as LTNP, indicating the need for larger, more definitive studies [13,16]. Most crucially, most studies have employed non-uniform definitions of LTNP, including those among adult populations, which makes comparisons with other populations difficult [15]. As a result, the generalizability of results from LTNP studies in adults to children is limited and compounded by the perceived limitations of pediatric studies - small available sample sizes, limited follow-up time, and a lack of consensus definitions of LTNP [15]. Therefore, there is very limited data on pediatric LTNP following vertical infection, especially among African populations with the highest disease burden [1]. Here, we leverage historical electronic medical record (EMR) data from two large clinical centers in Botswana and Uganda to determine the prevalence of pediatric LTNP and identify factors associated with progression.

## Materials and methods

### Study population

The Baylor International Pediatric AIDS Initiative (BIPAI) network is an open, prospective cohort of children infected with HIV initiated in 2001 with nine centers of excellence, six in Africa, one European, and two South American. This study considered participants enrolled by the Botswana-Baylor and Baylor-Uganda Children’s Clinical Centers of Excellence (COEs, Figure S1), the original centers of BIPAI, and the founding clinical centers for the Collaborative African Genomics Network (*CAfGEN*). *CAfGEN* is a multi-disciplinary, multi-institutional, inter-and intra-country network of African and American scientists, clinicians, and researchers. The principal aim of *CAfGEN* is to utilize genomics and related approaches to study gene-environment interactions in HIV/AIDS and its comorbidities among diverse African pediatric populations. This study was approved by Research Ethics Committees in Uganda, Botswana, and the USA.

### Study design

From mid-2003, children aged 0–18 who were confirmed to be vertically infected with HIV were prospectively enrolled in care at the COEs. For older children, the potential for vertical HIV transmission was ascertained by the history of maternal HIV infection or death due to HIV-related causes (excluding all those with self-reported sexual activity). It should be noted that in both countries, early infant diagnosis of HIV programs with polymerase chain reaction testing were initiated between 2005 (Botswana) and 2006 (Uganda). Socio-demographic and clinical characteristics from case report files are documented in the BIPAI EMR, and data entry clerks independently validate the data accuracy. At enrollment, an extensive clinical evaluation is undertaken to establish the prior medical history that guides proper World Health Organization (WHO) HIV staging. Follow-up visits are scheduled monthly for the first 3 months and quarterly after that to evaluate progression and guide ART initiation according to national ART guidelines. Additionally, community health liaisons maintain regular telephone contact with the participant families. For this analysis, we pooled data from eligible children enrolled in care up to the official censoring date of May 31, 2014. Therefore, we censored all children who had not progressed or initiated on ART. Only children who were ART naïve at enrollment were included in the time-to-progression analysis. This study adheres to the STROBE reporting guidelines for observational studies [17].

Demographic data collected included sex, dates of birth and enrollment, country, loss-to-follow-up (LTFU), transfers between clinics, and death. Death was determined following a home visit or proxied to the last known date alive if not otherwise defined as LTFU. Clinical data included weight, height (or length), and WHO clinical stage at enrollment in care. Although CD4^+^ T-cell (CD4) testing became available in 2002, it was only intermittently performed (i.e. semiannually) after 2010. Therefore, the earliest (i.e. within 6 months of enrollment) recorded CD4 count and percentage before ART were utilized as a baseline. Due to cost and the general lack of infrastructure in both countries at the time of setting up the COEs, pre-ART viral load testing was not readily available for this cohort except for those enrolled in specific studies. Weight-for-age (WFA), length/height-for-age (HFA), and body mass index-for-age (BFA) *z*-scores were computed according to WHO Child Growth Standard [18]. The WHO *z*-scores below two standard deviations of the population normal WFA, HFA, and BFA categorize undernutrition as underweight, stunted, and severely malnourished, respectively. The cohort was divided into three enrollment categories (pre-2006, 2006–2010, and post-2010 cohorts) based on the implementation of new national and WHO ART guidelines. The principal difference between the treatment guidelines was changes in the CD4 thresholds for starting ART from <200 cells/ml pre-2006 to ≤350 cells/ml 2006–2010 cohort. Also, 2010 heralded the universal recommendation to start children under 3 years on ART irrespective of their CD4 status. Given the retrospective nature of the study cohort, LTNP was defined conservatively as ART-naive asymptomatic HIV infection or having a CD4 count >500 cells/ml (or CD4% ≥25%) where available for at least 10 years [13,15]. To distinctly differentiate between long-term survivors and rapid progressors with reduced ambiguity, we used the age cutoff to 10 years. Rapid progression was defined as either two or more CD4% <15%, an AIDS-defining illness (WHO stage III or IV) or ART initiation within 3 years of birth (infection) [4]. For the purpose of deriving factors associated, we defined progression as ART initiation according to HIV treatment guidelines [19]. The date of birth serves as the origin or start time, while the date of progression or censoring marks the end times in the analysis.

### Statistical analyses

Baseline characteristics at enrollment were summarized descriptively for the entire cohort and compared between countries. Continuous data were summarized as median (IQR) and compared using the Wilcoxon rank–sum test. Categorical data were described using frequencies and compared by chi-square test between countries. The analysis’s primary outcome was time-to-progression, defined as either ART initiation (according to prevailing WHO HIV/AIDS treatment guidelines) or an AIDS-defining illness, whichever occurred first. The median and 10-year progression-free survival were estimated using the parametric survival model under the Weibull distribution due to a high rate of LTFU in the entire cohort. In contrast, we used the Kaplan-Meier method to estimate the rate of loss of LTNP status.

Competing risk regression analysis was used to estimate the subdistribution hazard ratios for the candidate predictors of progression accounting for LTFU as a competing event. All factors with *P*-value <0.2 at univariate analysis were entered into a multivariate regression model, except CD4 counts which is a clinical measure of progression, and HIV viral load measurements, which were only available for 847 participants. We accounted for clinically significant interaction terms and selected the final model with the lowest Bayesian Information Criterion. We considered a two-tailed *P*-value <0.05 as statistically significant. We used STATA17 and R statistical software v4.0.3 for all analyses.

## Results

### Baseline characteristics

**Figure 1.**
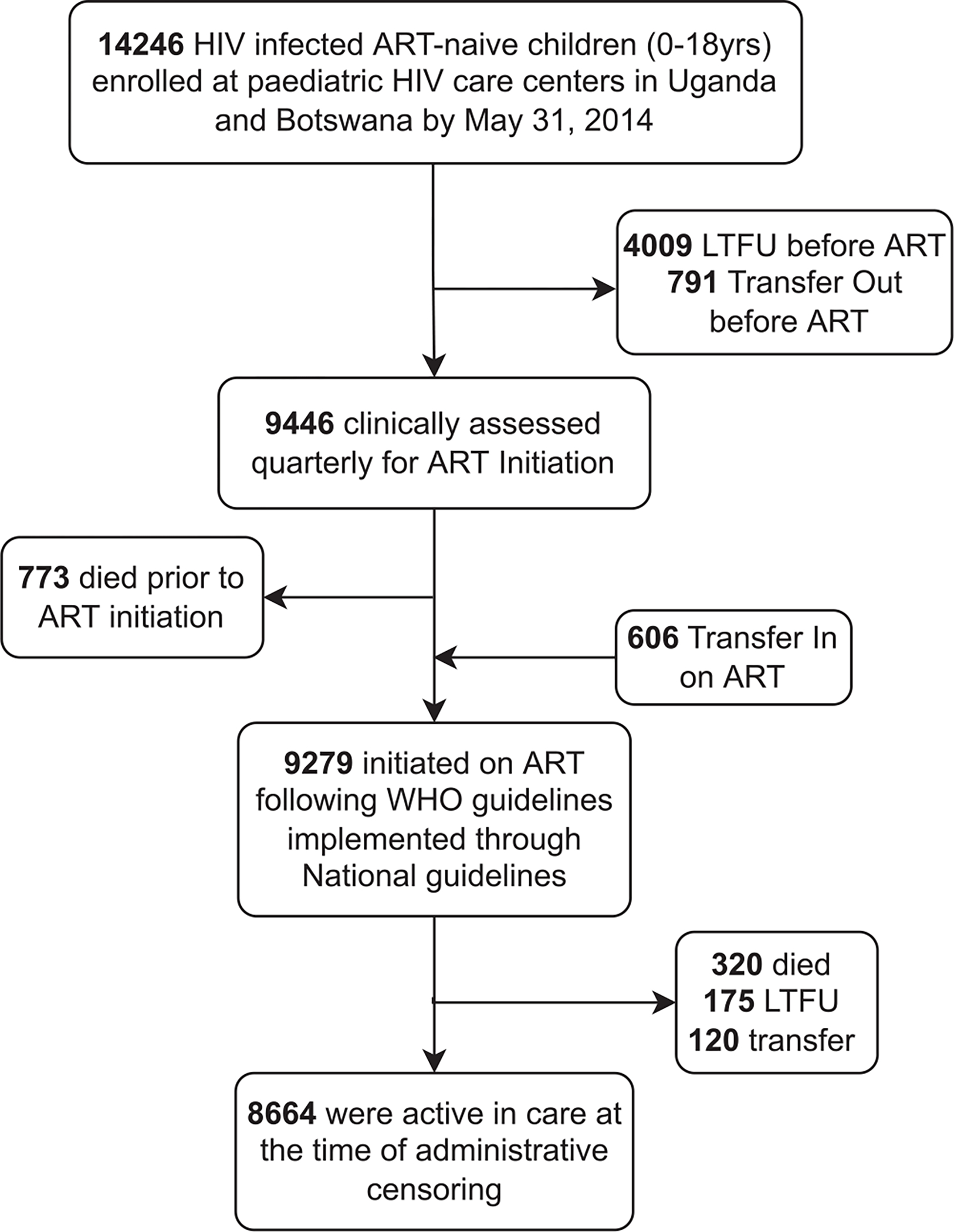
The flow of study participants into HIV care. Abbreviations: ART, antiretroviral therapy; LTFU, loss-to-follow-up.

Overall, 15,124 children were enrolled in care between 2002 and 2014, 14,246 of whom were ART naïve at baseline and were included in this analysis (Figure 1). More than half of the children were female (51%), with a similar sex distribution in Botswana and Uganda (Table 1). Most participants (58%) were in care by the age of 5 years, with a median age at enrollment of 3.7 years (IQR 1.1–8.4); however, children in Botswana tended to be older at enrollment than in Uganda (5.3 years; IQR: 2.0–10.8 vs 3.5 IQR: 1.0–8.4 in Uganda; *P* <0.0001; Table 1). Most children in Botswana presented with advanced disease, and as a result, the median age at initiating ART was lower in Botswana than in Uganda (Table 1, Figure S2). We found potential differences in the median CD4 count, HIV RNA viral load, LTFU mortality, and BFA between the two countries. However, the distribution of the baseline HIV RNA viral load, WFA, and HFA were similar (Table 1).

### Proportion of LTNP among pediatric HIV populations in Africa

Selecting those children who were ART naïve at enrollment resulted in 14,246 (94%) participants, the majority of whom (12,679; 89%) were from Uganda (Figure S3). This large difference between the countries is not unexpected, given the population sizes of Uganda (pop. 47.12 million; June 2022; World Population Prospects 2019) and Botswana (pop. 2.35 million; June 2022; World Population Prospects 2019). The overall median progression-free survival was 6.3 years (95% confidence interval [CI]: 6.1–6.4, Figure S4a; Uganda 6.5 years vs Botswana 4.7 years Figure S4b), while the 10-year progression-free survival was 29.4% and varied between Botswana and Uganda (20.1% vs 30.1%, Figure S4b). The 14,246 children studied contributed 84,475 person-years of infection. Among this group, 892 children were identified as LTNP, indicating a 6.3% prevalence (Table S1). At the other end of the continuum, 28.4% (4,044) were rapid progressor. In Botswana, we found a 9.5% LTNP prevalence, which was higher than Uganda’s 5.9%, which might be consistent with Botswana’s older median age at enrollment in care (Table S1). We then used a Kaplan-Meier estimate to examine the time progression from LTNP status. The median time to loss of LTNP status was 13 years (95% CI 12.7–13.1; Figure S4c). The loss of LTNP status was statistically faster among children in Botswana compared to Uganda (*P* = 0.0001; Figure S4d). Overall, the progression-free survival among LTNPs was 22.8% (95% CI 20.2–25.7) at 15 years and 1.1% (95% CI 0.6–2.1) at 20 years following vertical HIV infection.

### Risk factors of HIV progression among children

**Figure 2.**
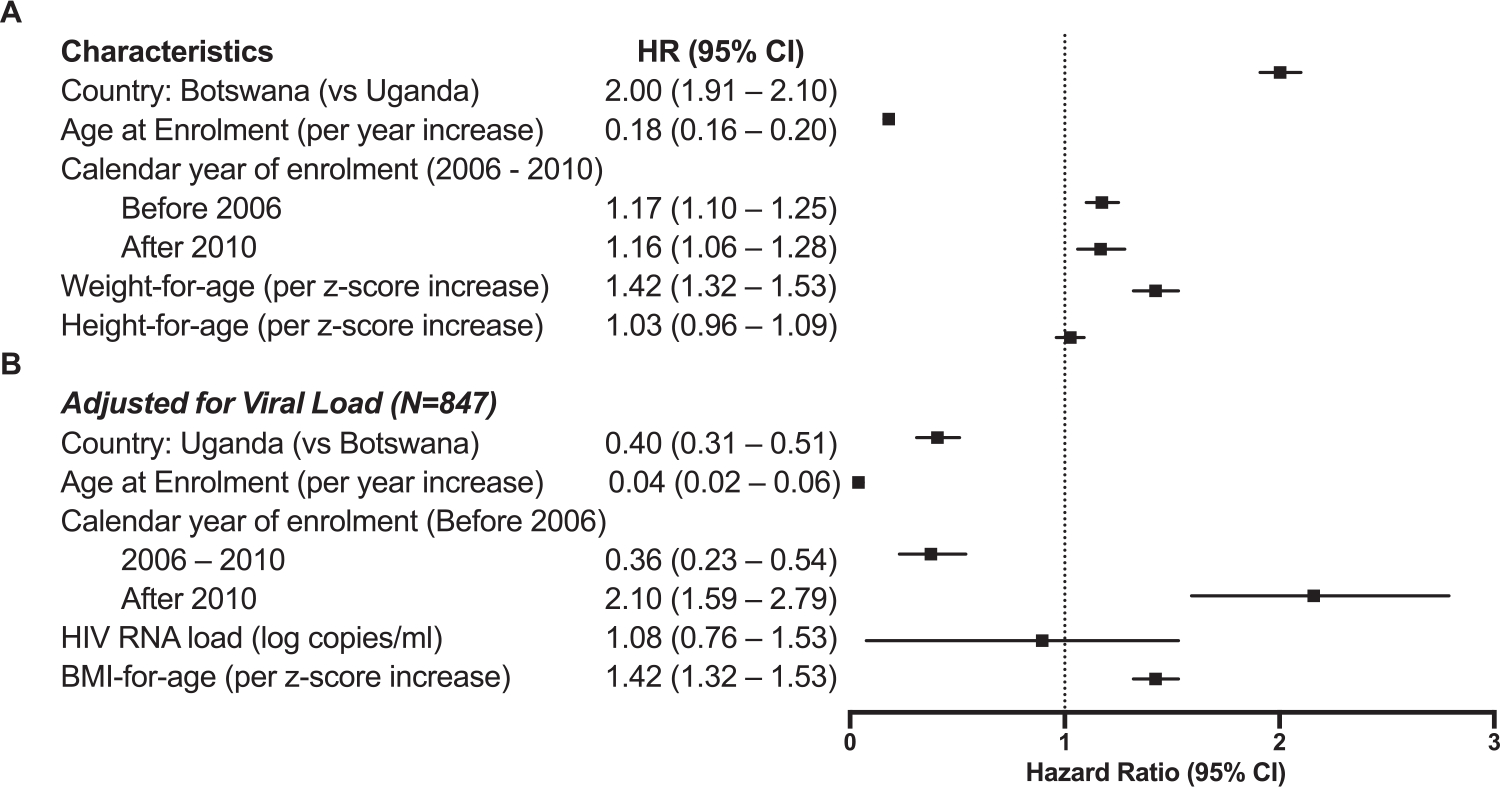
Adjusted hazard ratios of progression without n = 10,078 (a) and with n = 847 (b) HIV viral load in the model. Abbreviations: BMI, body mass index; CI, confidence interval; HR, adjusted sub-distribution hazard ratio.

**Figure 3.**
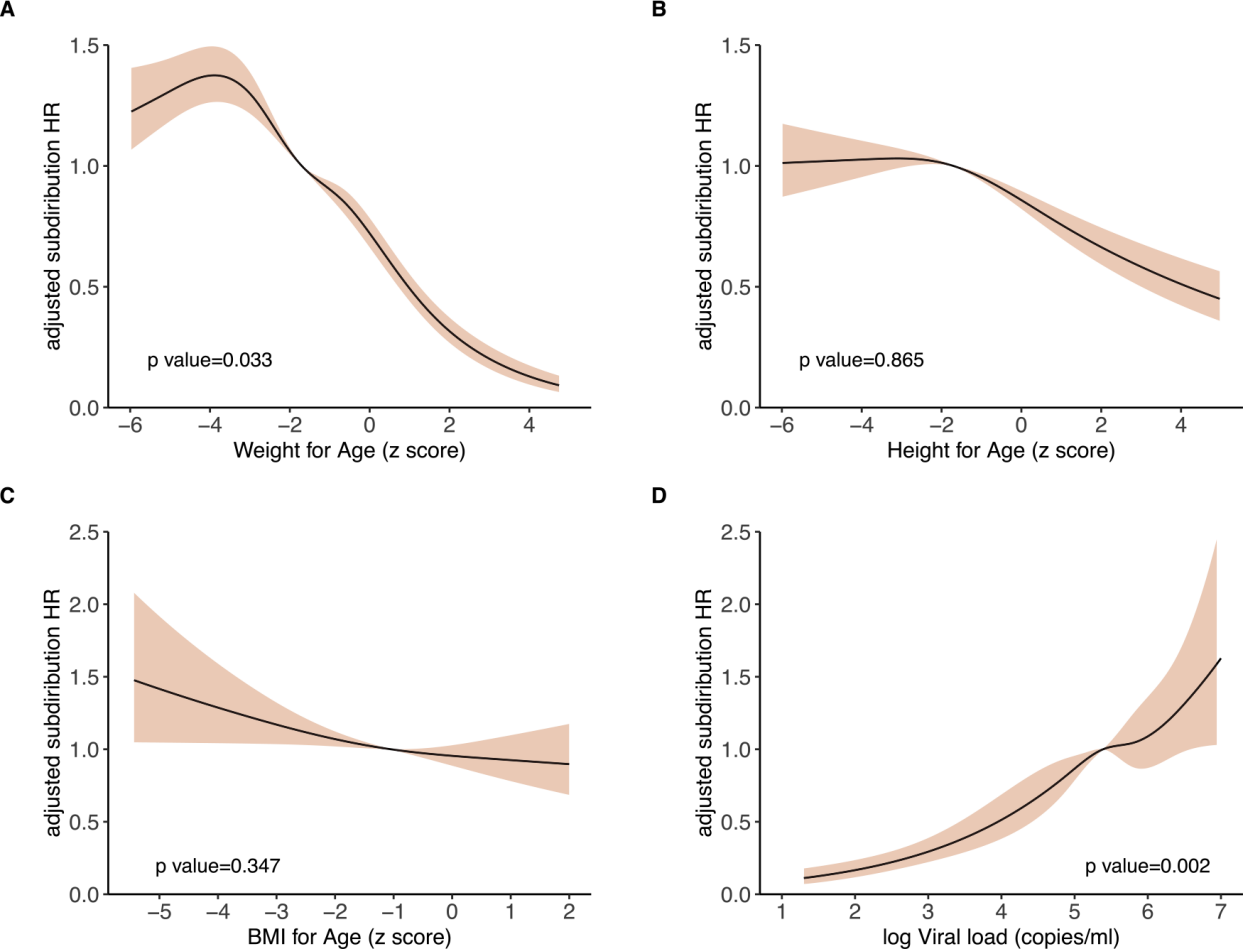
Association of weight-for-age (a), height-for-age (b), BMI-for-age (c) and HIV viral load (c) with progression. The solid black line represents the fitted line of the association between the predictors and the estimated hazard ratio of progression. The shaded area represents the 95% confidence interval. For the upper panel the model was adjusted for country of origin, age at enrollment, year of enrollment, weight-for-age and height-for-age. For the lower panel the model was conducted in a subset of children with known viral load measurements at enrollment and was adjusted for country of origin, age at enrollment, year of enrollment, BMI-for-age and HIV RNA viral load. Cubic splines with five and three knots were used to adjust age at enrollment as well as weight-for-age and height-for-age to better fit the data. Abbreviations: BMI, body mass index; HR, hazard ratio.

Having established the proportion of LTNP among children, we then examined the predictors of progression on enrollment into care. Here we excluded 4,168 children with incomplete clinical records and examined the relationship between progression with multiple factors, including country, sex, age at enrollment, WFA, HFA, BFA, viral load, calendar year of birth, and calendar year of enrollment. In univariate analysis, male sex, country, age at enrollment, undernutrition (severe malnutrition, underweight, stunting), and being born after 2004 and enrolled after 2010 were associated with progression (Table 2). In multivariate analysis, underweight children at enrollment were independently associated with progression (adjusted subdistribution hazard ratio [aHR_sd_] 1.42, 95% CI 1.32–1.53; Table 2, Figure 2) after adjusting for age, country, and enrollment cohort, while accounting for those who died or were LTFU. Although not statistically significant, children who were stunted at enrollment were at a higher risk of progression (aHR_sd_ 1.03, 95% CI 0.96–1.09; Figure 3b) than normal statured children. Children from Botswana had a 2-fold increased risk of progression compared to Uganda (aHR_sd_ 2.00, [95% CI: 1.91–2.10]). We observed a significant “cohort effect” where children enrolled in care between 2006–2010 and after 2010 progressed faster to AIDS (aHR_sd_ 1.14 and 1.13 respectively; Table 2). In a subset of 847 children with viral load measurements within 6 months of enrollment, multivariate analysis found that low BFA and enrollment after 2010 were independently associated with progression (Table S2, Figure 2 and S4c). Additionally, a high HIV RNA viral load was associated with an increased risk of progression, although not statistically significant (Figure 3d).

## Discussion

This study presents results from one of the largest African pediatric HIV cohorts, covering approximately 8% and 12% of children living with HIV in Botswana and Uganda, respectively. We aimed to estimate the frequency of LTNP and examine the factors associated with progression in children using available demographic and baseline clinical data. We applied a definition of LTNP to include children without an AIDS-defining illness, no ART, or no more than one CD4 count below 500 cells/ml (or CD4% ≤25%) for 10 years since infection meeting our criteria [15]. Most demographic and baseline clinical characteristics differed among children from Botswana and Uganda, consistent with cultural and economic differences between countries/populations. However, once enrolled in care, national HIV care and treatment guidelines or programs were highly congruent, as implemented in the BIPAI network, to which both clinical centers subscribe. We, therefore, chose to consider the two populations together for outcome analyses.

Because there have been few studies on the prevalence of LTNP among children in Africa, our multiethnic study in a geographically and genetically diverse HIV background gives the first and largest account of an estimate of LTNP prevalence in Africa. Furthermore, this study takes advantage of a large number of children infected with HIV in a location where the disease burden remains very high. Previous research, especially in Western populations, reported conflicting rates of LTNP ranging from as low as 1.5 to 27% in substantially smaller populations and utilized varying age cutoffs (5 years, 8 years, or 10 years) to define LTNP [20,21]. We observed a frequency of LTNP that was in-between that reported in these studies; however, our study is 30 times larger than the largest known study in the USA, which included 422 children and utilized a similar clinical definition [13–15,20,21]. The heterogeneity of definitions for LTNP across studies may contribute to variations in their observed frequency compared to other populations, highlighting the need for standardized criteria in research [15]. Notably, a third of our cohort were LTFU or transferred out of the COEs and did not have recorded outcomes. Consequently, the actual proportion of LTNPs may differ from the estimates here; in Botswana, where the LTFU was significantly lower (5% vs 32% in Uganda), the frequency of LTNPs was higher (11% vs 7% in Uganda), although most of the children in Botswana were also enrolled at an older age. Given the asymptomatic nature of their presentation, it is plausible that LTNPs were present in these communities prior to the creation of pediatric HIV care clinics (survivor bias), although presenting late to clinic care creates some uncertainty around their LTNP status. Children in the COE were derived from Botswana and Uganda’s urban catchment areas in the capital city. Rural-to-urban migration meant that we had all districts represented in our study. To our knowledge, this is the first and largest study to examine the loss of LTNP status among African children. Compared with studies from adult populations, our population’s median loss of LTNP status is higher than in Western adult populations [22]. In a recent study of mixed-racial adult populations, the median time to loss of LTNP status was 12.5 years which is lower but comparable to 13 years in our study [4]. However, the median survival of 13 years in this study is slightly shorter but comparable to a study in Thai children where it was 13.7 years [10]. Notably, the Thai study employed a LTNP cutoff of 8 years, whereas our study used a 10-year cutoff [10]. These differences further highlight the value of harmonization of the definition of LTNP status. Our study further confirms that children, like adults, rapidly lose their LTNP status, with 50% progressing to AIDS within 3 years after 10 years of stable infection [4].

Children are expected to progress faster to AIDS than adults (e.g., <35% are progression-free by 2 years of infection) in part due to their developing immune system [14]. However, we unexpectedly found that half the children in our cohort remained progression-free for ≥ 6 years, and more than one-third for ≥ 10 years. A meta-analysis of 57 studies in mostly adult cohorts globally found the estimated 10-year progression-free survival to be 26%, much lower than in our cohort, especially among children from Uganda, which may be attributed to survival bias in pediatric cohorts [23]. Additionally, this study used a composite definition of progression-free survival as initiating ART regardless of the reason. We recognize that during the period under study, international and national guidelines on HIV treatment changed three times. The most significant changes affected young children, which could affect the progression-free survival estimate. However, it is anticipated that such changes should lower survival in this age group. The high progression-free survival may also be attributed to the impact of unmeasured confounding of perinatal administration of zidovudine and nevirapine monotherapy or combination therapy in prevention of mother-to-child transmission (PMTCT) programs, which has been shown to delay HIV progression [5,24–26]. Speculatively, the disparity in progression-free survival between the two countries could be accounted for by the differences in the HIV epidemiological curves in Botswana and Uganda. Early implementation of strategies such as cotrimoxazole prophylaxis against opportunistic pathogens significantly reduces the risk of progression, especially early in infancy (in our study, we found that 60% of children in Uganda were enrolled below 5 years and received the prophylactic intervention) [11,27]. Therefore, the estimates provided here may overestimate progression-free survival, which would be expected in an observational cohort compared to prospective cohorts.

There exists a contributory relationship between HIV progression and undernutrition. HIV increases the risk of undernutrition due to higher metabolic demands and dysregulation, and conversely, undernutrition can influence immune dysfunction, which accelerates progression [28]. Predictably, the proportion of undernutrition at baseline in our cohort was higher than reported for non-HIV-infected children in African populations but similar to estimates among HIV-positive cohorts from Africa [12]. In our cohort, it is challenging to determine the direction of the relationship; however, there is a possibility that these children experience bidirectional effects of HIV on nutrition and vice versa, leading to an excess of undernutrition [28]. Studies in pregnant women infected with HIV provide evidence of intrauterine growth retardation that could explain the persistent undernutrition even in older children infected with HIV. However, other factors such as food insecurity and frequent childhood illnesses (respiratory tract infections and diarrhea) may have a role play [29,30].

Furthermore, while many studies have reported an increased risk of disease progression and mortality in males, but the sex effect lacks consistent statistical significance [4,31]. This inconsistency may be attributed to the heterogeneous distribution of other risk factors, including CD4 counts, WHO stage, and host genetic variations. Nevertheless, previous studies from Uganda and Botswana have demonstrated that males exhibit significantly higher viral loads compared to females, potentially contributing to accelerated disease progression and increased mortality [32,33]. This is consistent with our study, where we observed a similar trend, with males showing a slightly higher mean viral load than females (5.0 vs 4.9 log10 copies/ml).

The distribution of HIV clades differs between Botswana and Uganda, and progression has been associated with these differences, albeit with some inconsistency in direction and effect [11,34,35]. We observed a higher proportion of rapid progressors in a predominantly HIV-1 clade C epidemic in Botswana compared to a population with equal distribution of HIV 1 clades A or D and their recombinant clade AD in Uganda [34]. Our findings generally contradict prior research in this area. Amornkul reported faster progression to AIDS with subtype D vs A (HR 1.94, *P* = 0.0006) and C vs A (HR 1.60, *P* = 0.01) [34,35]. Similarly, a study among women found that Zimbabweans infected with HIV-1C had a 2.5-fold slower CD4 T-cell decline compared to Ugandans infected with HIV-1A or D [11]. Therefore, in our cohort, this difference could be explained by the lower median anthropometric *z*-scores and CD4 counts at baseline among children in Botswana compared to those in Uganda (Table 1) [34]. Furthermore, Botswana and Uganda are at different stages in the HIV epidemic transition (epidemic curve) [36]; Uganda is estimated to have reached the peak of HIV-related deaths in 1998 compared to Botswana, which reached the highest number of HIV-related deaths in 2002 [36]. We were also able to detect a “cohort effect” with a high likelihood of progression among children enrolled after 2010, which could be a consequence of the increased number of younger children more recently enrolled at the COEs or changes in ART treatment guidelines. Incidentally, a few years earlier, the COEs started implementing programs linked to PMTCT services where HIV-exposed infants were increasingly recruited after birth to assess possible vertical infection. Furthermore, the cohort effect arises from individuals in the same recruitment cohort that will encounter similar events at comparable ages. Birth and enrollment cohorts are exposed to varying events at different life stages and experience distinct levels of economic, behavioral, policy (ART), and environmental risks of progression. Additionally, the observed cohort effect is also predicted to affect and skew LTNP estimates. The children in this study were born from 1986, the pre-ART era to 2014, when ART was widely available; however, we did not find a “calendar effect,” which has not been invariably associated with progression [4].

There are some limitations to this study. The results are potentially biased toward the Ugandan population, contributing to the vast majority (89%) of the children analyzed. However, this is not unexpected given the population sizes in both countries; 47.12 million in Uganda compared to 2.35 million in Botswana, although in a relatively random sub-study of the 847 children with HIV RNA load measurements, we found similar trends (Table S2). Previous studies have reported a significant mortality rate, with over 50% of deaths within the first 2 years if no ART is initiated [5]. As our study focuses on LTNP it is important to acknowledge that the high early mortality rate might introduce survival bias, as those who die early due to the infection would not be included in our analysis. This survival bias could potentially lead to an imprecise estimation of the prevalence of LTNP, as our study population may be enriched with individuals who have survived beyond the early critical period. Additionally, the shifting WHO ART recommendations from 2003, 2006, and 2010 can significantly impact LTNP calculations and estimation of progression-free survival. For example, in 2008, the WHO advised that all infants infected with HIV under 24 months be started on antiretrovirals (ARV), regardless of clinical or immunological status [37]. However, the BIPAI clinics did not adopt this guideline until 2010 (consequently, all these children are counted as progressors), which may have had a minor impact on our LTNP status because no children would have survived 10 years by the censoring date of our study. Nonetheless, with validation from other pediatric cohorts in African populations, this study finds that our findings provide a robust estimate of LTNP and confirm the evidence for integrating nutritional programs in pediatric HIV care to reduce early mortality [38]. We intended to examine a broader range of both demographic and clinical factors previously associated with progression in children; [14,20], (e.g. maternal inutero factors, maternal viral load, birth weight, prematurity, antenatal care, viral clade, neuro psychomotor development, immunological profiles, psychosocial assessments, cognitive impairment); however, most of the desired data was either not routinely collected, not available within 6 months of enrollment into care, or was otherwise unavailable. This is likely the result of the piecemeal HIV care and treatment guidelines and the fractured healthcare delivery systems in Africa. Therefore, our analysis was limited to a candidate set of factors within the broader aims of CAfGEN. Nevertheless, we demonstrate a significantly increased risk of progression among children from Botswana, undernutrition (underweight and stunted) and those who were enrolled before 2006 or after 2010.

## Conclusion

With the advent of test and treat strategy to reduce HIV-associated morbidity and mortality, the future identification of LTNP individuals is impossible. Progression-free survival as conservatively defined here in African pediatric HIV populations is more prolonged than previously recognized; however, the frequency of LTNP is low. Undernutrition and ethnicity are independently associated with progression in children. Finally, extended phenotyping of this pool of children identified as LTNP can support studies based on extremes of disease progression to understand genetic and immunologic mechanisms of natural control of HIV.

## Supplementary Material

1

2

3

4

5

6

7

## Figures and Tables

**Table 1:** Comparison of demographic and clinical characteristics of children in Botswana and Uganda.

Variable	Overall	Country		*P*-value
		Uganda	Botswana

No. of Children	14246	12709 (89)	1537 (11)	
Sex, *n* (%)				0.144
Female	7295 (51)	6535 (51)	760 (49)	
Male	6951 (49)	6174 (49)	777 (51)	
Age at enrollment, median (IQR) yrs	3.7 (1.1–8.4)	3.5 (1.0–8.1)	5.3 (1.3–9.4)	<0.001
<2	5250 (36.8)	4761 (37.4)	489 (31.8)	<0.001
2 to <5	2967 (20.8)	2722 (21.4)	245 (15.9)	
5 to <10	3375 (23.7)	2909 (22.9)	466 (30.3)	
10 to 18	2654 (18.6)	2317 (18.2)	337 (21.9)	
Year of birth, *n* (%)				<0.001
≤2004	8864 (62)	7654 (60)	1210 (79)	
>2004	5382 (38)	5055 (40)	327 (21)	
Year of enrollment, *n* (%)				<0.001
<2006	5099 (36)	4344 (34)	755 (49)	
2006–2010	6537 (46)	6027 (48)	510 (33)	
>2010	2610 (18)	2338 (18)	272 (18)	
World Health Organizations stage, *n* (%)				<0.001
I or II	4853 (49)	4393 (51)	460 (34)	
III	3257 (33)	2746 (32)	511 (38)	
IV	1818 (18)	1457 (17)	361 (27)	
Anthropometric measurement, median (IQR)^a^				
Weight-for-age, Z-score	−1.74 (−3.19 −0.43)	−1.7 (−3.2 - −0.3)	−1.8 (−2.97 – −0.7)	0.043
Height-for-age, Z-score	−1.76 (−2.89 – −0.55)	−1.7 (−2.9 – −0.5)	−1.6 (−2.6 – −0.6)	0.055
Body mass index-for-age, Z-score	−0.83 (−2.06 – 0.16)	−0.80 (−2.03 – 0.19)	−1.08 (−2.19 – −0.24)	<0.001
CD4 count (cells/ml)^a,b^	309 (101–597)	302 (90–603)	329 (159–559)	0.075
CD4%, median (IQR)^a,c^	17.0 (11.0–24.0)	17.0 (11.0–24.0)	19.2 (12.0–27.0)	<0.001
HIV RNA load (log10 copies/ul)^a^	5.4 (4.8–5.8)	5.6 (4.9–5.8)	5.3 (4.7–5.7)	<0.001
Age at antiretroviral therapy initiation, median (IQR) yrs	6.3 (2.1–11.0)	6.4 (2.3–11.2)	5.7 (1.6–10.1)	<0.001
Below 5	3725 (43)	3 0 73 (43)	652 (45)	<0.001
5 to < 10	2313 (27)	1882 (26)	431 (29)	
10 to 18	2626 (30)	2248 (31)	378 (26)	
Clinical outcome, *n* (%)				
Alive	7998 (56)	6794 (53)	1204 (78)	
Loss-to-follow-up	4210 (30)	4126 (33)	84 (6)	
Transfer out	911 (6)	911 (7)	0 (0)	
Death	1127 (8)	878 (7)	249 (16)	

Abbreviations: CD, clusters of differentiation; IQR, interquartile range; SD, standard deviation; yrs, years.

*P*-values compare the distribution of each variable by country.

a The data available for the variable is less than the total number of participants analyzed due to missing values.

b For children above 60 months.

c For children below 60 months.

Note: All measurements were done at enrollment

**Table 2:** Subdistribution hazard ratios of progression from competing risk models.

Characteristics	Univariate HR_sd_ (95% CI)	*P*-value	Multivariate aHR_sd_ (95% CI)	*P*-value

Country		<0.001		<0.001
Uganda	1		1	
Botswana	1.44 (1.36 – 1.52)		2.00 (1.91 – 2.10)	
Sex				
Female	1	<0.001		
Male	1.19 (1.14 – 1.24)			
Age at Enrollment (per 1-year increase)	0.20 (0.18 – 0.22)	0.011	0.18 (0.16 – 0.20)	0.001
Year Birth				
Before 2004	1	<0.001		
After 2004	3.45 (3.22 – 3.70)			
Year of enrollment				<0.001
Before 2006	1		1	
2006–2009	0.96 (0.91 – 1.01)	0.14	1.14 (1.07 – 1.22)	
After 2010	1.19 (1.12 – 1.26)	<0.001	1.13 (1.02 – 1.24)	
HIV RNA load, (log10 copies/*μ*l)^a^	1.89 (1.52 – 2.30)	<0.001		
Weight-for-age Z-score (per Z increase)	0.68 (0.64 – 0.72)	0.031	0.77 (0.68 – 0.87)	<0.001
−1.75	1		1	
−3.75	1.24 (1.16 – 1.34)		1.42 (1.32 – 1.53)	
Height-for-age z-score (per Z increase)	0.86 (0.83 – 0.88)	<0.001	1.00 (0.92 – 1.08)	0.91
−1.75	1		1	
−3.75	1.22 (1.17 – 1.27)		1.03 (0.96–1.09)	
BMI-for-age z-score (per Z increase)^b^	0.79 (0.76 – 0.82)	<0.001		
−0.85	1			
−2.85	1.32 (1.28 – 1.38)			

Abbreviations: aHR_sd_, adjusted subdistribution hazard ratio; BMI, body mass index; CI, confidence interval.

N = 10078

a n = 847

b BMI-for-age was not included in any model with weight-for-age or height-for-age.
