# Supplementary material for: Long-term non-progression and risk factors for disease progression among children living with HIV in Botswana and Uganda: A retrospective cohort study
Source: Int J Infect Dis. Author manuscript; Available in PMC 2024 Feb 5. (PMC10843817; doi:10.1016/j.ijid.2023.11.030)
Supplement: 1 [file NIHMS1958773-supplement-1.docx]

LEGENDS:

Figure S1. Map showing the location of the Clinical Centers of Excellence in Botswana and Uganda.

Figure S2. Distribution of age of progression in the entire cohort (A) and in Uganda vs. Botswana (B). The dashed lines in Fig. S1B indicate the median age at progression in the two countries.

Figure S3. Number of children enrolled per year in Botswana and Uganda. Enrolment into care at the Baylor Pediatric HIV Clinical Center of Excellence started in 2002 and 2003 in Botswana and Uganda, respectively.

Figure S4. Weibull parametric estimate of Progression-free survival among the total number of children (A) and children in Botswana and Uganda (B). Kaplan-Meier estimate of progression-free survival after attaining LTNP status (C) and (D) children in Botswana vs. Uganda. The shaded area indicates the 95% confidence interval. .
