# Supplementary figures and images for: Long-term non-progression and risk factors for disease progression among children living with HIV in Botswana and Uganda: A retrospective cohort study
Source: Int J Infect Dis. Author manuscript; Available in PMC 2024 Feb 5. (PMC10843817; doi:10.1016/j.ijid.2023.11.030)

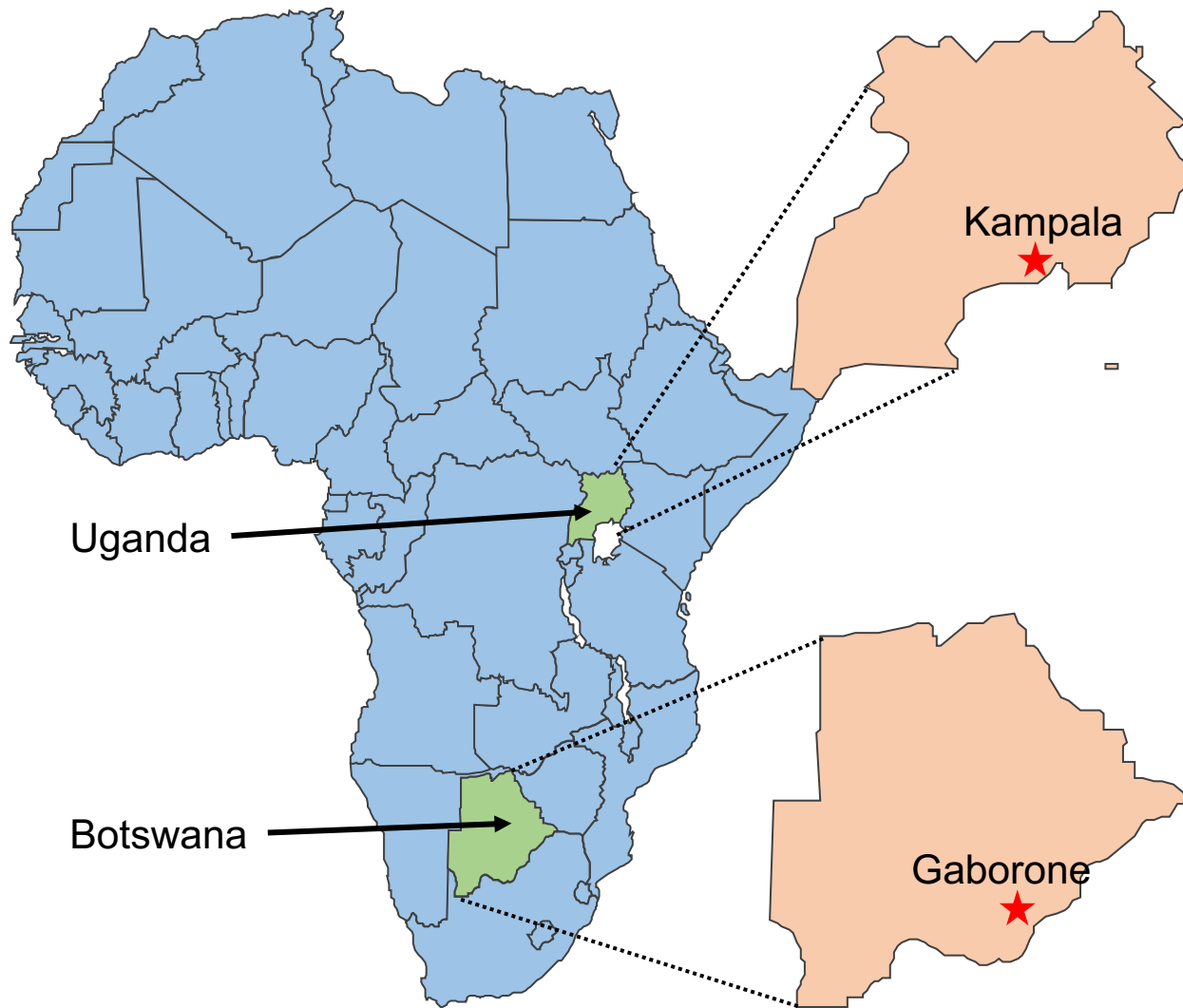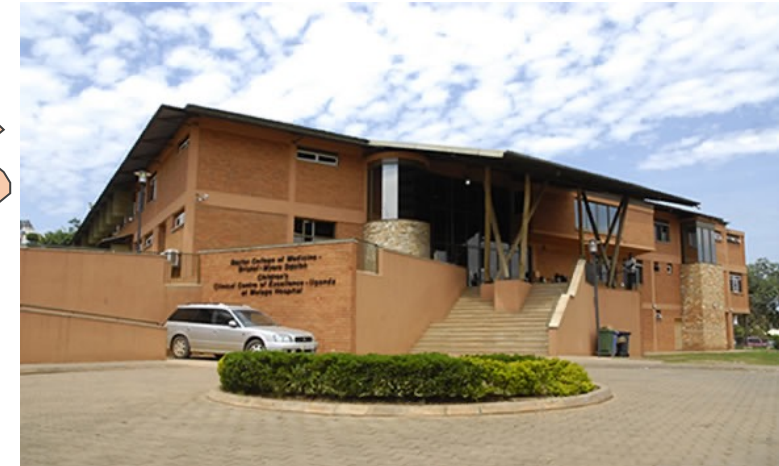

Baylor-Uganda Children's Clinical COE

Botswana-Baylor Children's Clinical COE

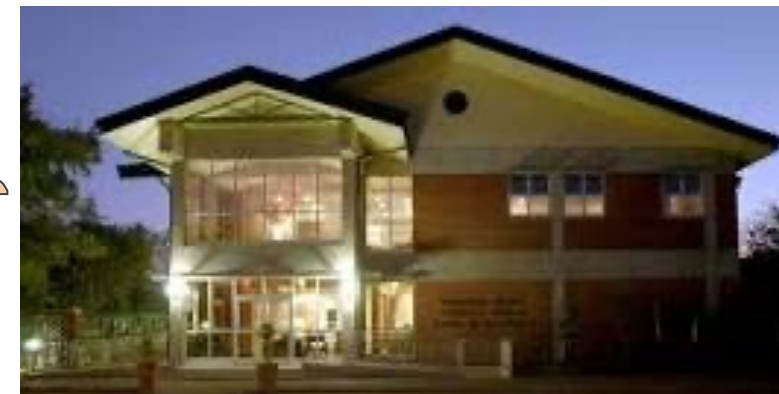

Supplement: 2 [file NIHMS1958773-supplement-2.pdf]

**A**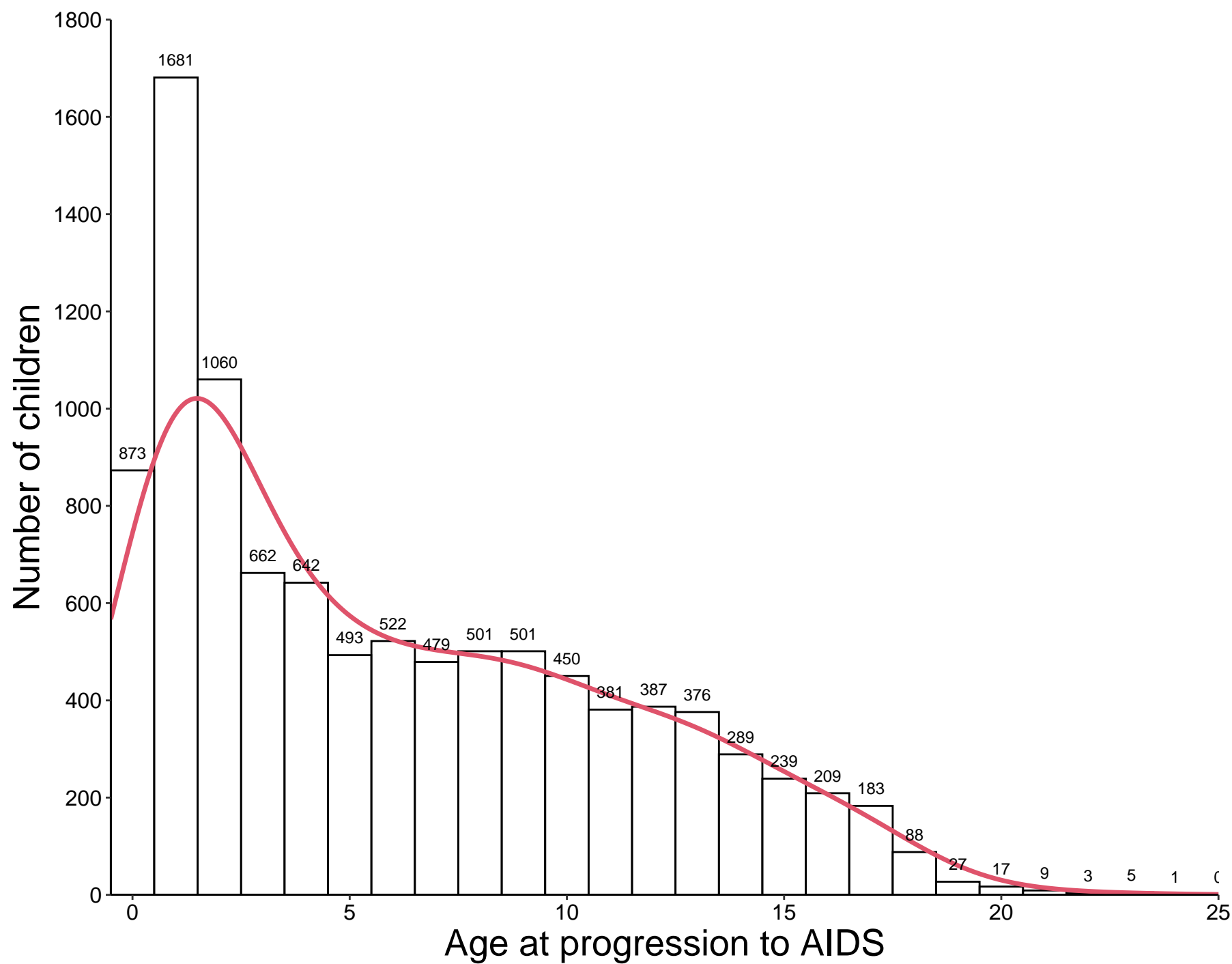**B**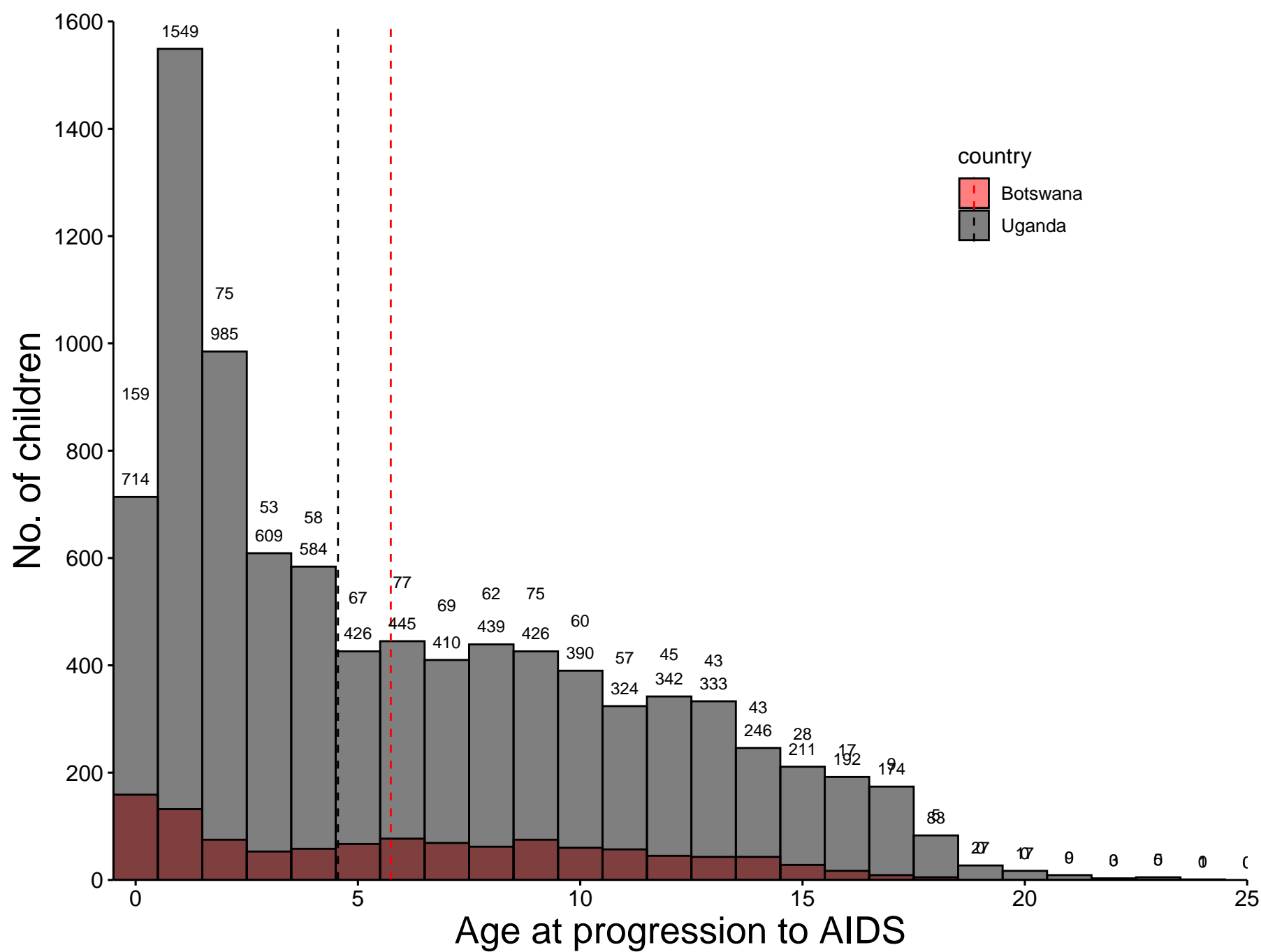

Supplement: 3 [file NIHMS1958773-supplement-3.pdf]

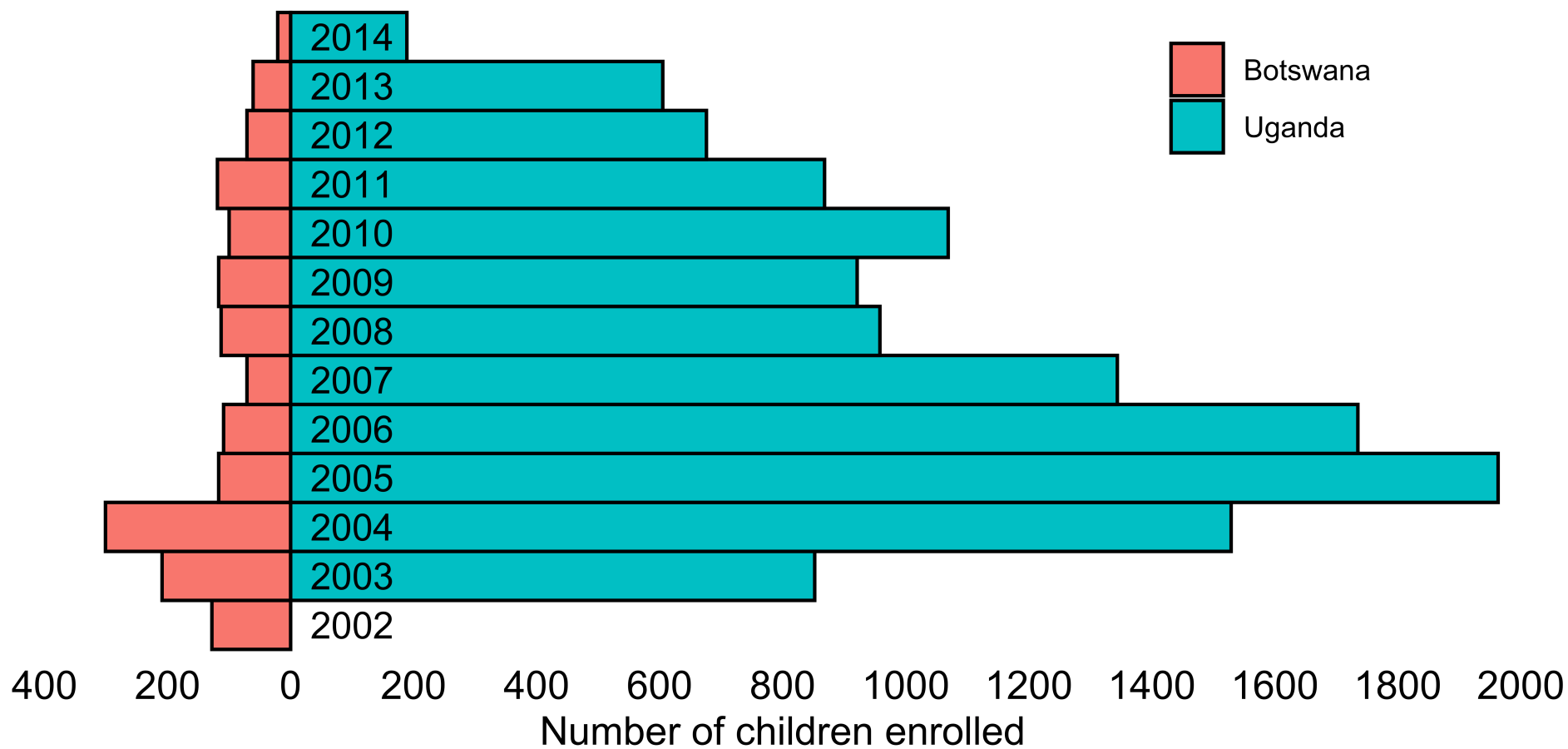

Supplement: 4 [file NIHMS1958773-supplement-4.pdf]

A

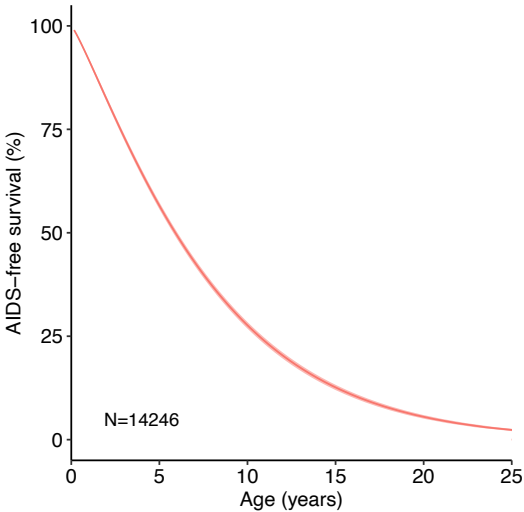

B

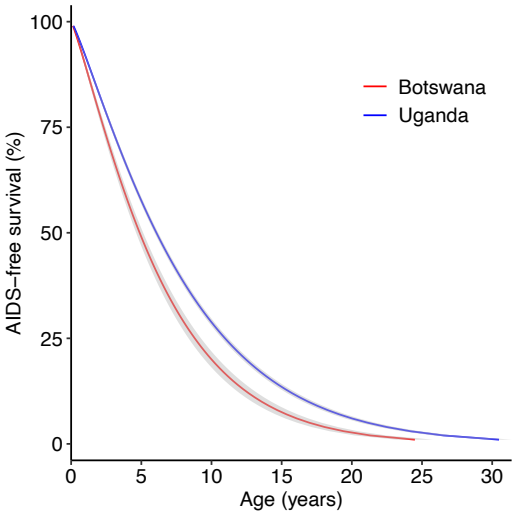

A

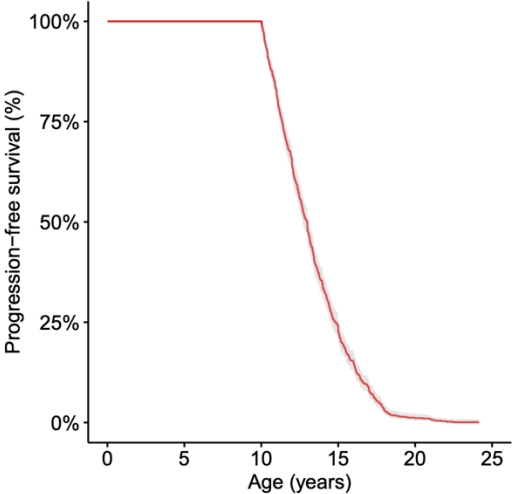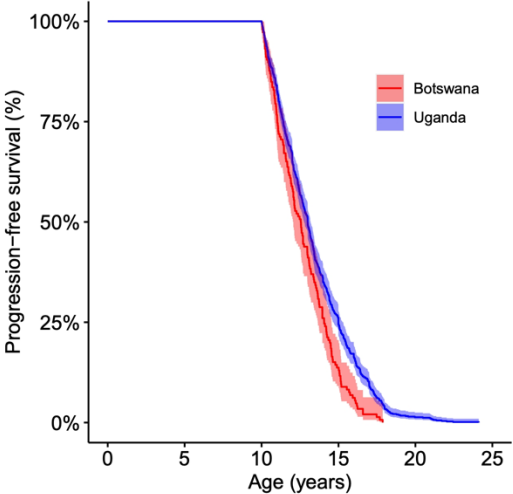

Supplement: 5 [file NIHMS1958773-supplement-5.pdf]
