# Supplementary material for: Long-term non-progression and risk factors for disease progression among children living with HIV in Botswana and Uganda: A retrospective cohort study
Source: Int J Infect Dis. Author manuscript; Available in PMC 2024 Feb 5. (PMC10843817; doi:10.1016/j.ijid.2023.11.030)
Supplement: 6 [file NIHMS1958773-supplement-6.docx]

| **Table S1:** Comparison of demographic and clinical characteristics of LTNP and Progressors | | | | |
| --- | --- | --- | --- | --- |
| **Variable** | | **Category** | | **p–value** |
|  |  | **LTNP** | **Progressors** |  |
| No. of Children | | 892 (89) | 13354 (11) |  |
| Country, *n (%)* | |  |  | <0.001 |
|  | Uganda | 746 (83.6) | 11963 (89.6) |  |
|  | Botswana | 146 (16.4) | 1391 (10.4) |  |
| Sex, *n (%)* | |  |  | <0.001 |
|  | Female | 531 (59.5) | 6764 (50.7) |  |
|  | Male | 361 (40.5) | 6590 (49.3) |  |
| Age at Enrolment, mean (SD) yrs | | 11.25 (3.70) | 4.79 (4.52) | <0.001 |
|  | <5 | 59 (6.6) | 8158 (61.1) | <0.001 |
|  | 5 to <10 | 209 (23.4) | 3166 (23.7) |  |
|  | 10 to 18 | 624 (70.0) | 2030 (15.2) |  |
| Year of birth, *n (%)* | |  |  | <0.001 |
|  | ≤2004 | 892 (100) | 7972 (59.7) |  |
|  | >2004 | 0 (0) | 5382 (40.3) |  |
| Year of enrolment, *n (%)* | |  |  | 0.004 |
|  | <2006 | 295 (33.1) | 4804 (36.0) |  |
|  | 2006—2010 | 456 (51.1) | 6081 (45.5) |  |
|  | >2010 | 141 (15.8) | 2469 (18.5) |  |
| WHO Stage, *n (%)* | |  |  | <0.001 |
|  | I or II | 537 (63) | 4354 (47.7) |  |
|  | III | 208 (24.4) | 3049 (33.5) |  |
|  | IV | 107 (12.6) | 1711 (18.8) |  |
| Anthropometric measurement, mean (SD) | |  |  |  |
|  |  |  |  |  |
|  | Weight–for–age, Z–score* | –0.89 (2.21) | –1.83 (2.20) | <0.001 |
|  | Height–for–age, Z–score* | –0.76 (2.34) | –1.61 (2.13) | <0.001 |
|  | BMI–for–age, Z–score* | –0.73 (1.50) | –1.09 (1.74) | <0.001 |
| CD4 count (cells/ml), mean (SD)§* | | 850.08 (368.66) | 683.11 (590.11) | <0.001 |
| CD4%, mean (SD)†* | | 31.35 (6.55) | 18.01 (9.39) | <0.001 |
| HIV RNA load (log10 copies/uL)* | | 4.20 (1.39) | 5.22 (1.10) | <0.001 |
| **Abbreviations:** yrs – years, SD – standard deviation. p–values compare the distribution of each variable by country. *the data available for the variable is less than the total number of participants analyzed due to missing values. | | | | |
|  |  |  |  |  |
|  |  |  |  |  |
| § for children above 60 months, † for children below 60 months | | | | |
| Note: All measurements were done at enrolment | | | | |
