# Supplementary material for: Long-term non-progression and risk factors for disease progression among children living with HIV in Botswana and Uganda: A retrospective cohort study
Source: Int J Infect Dis. Author manuscript; Available in PMC 2024 Feb 5. (PMC10843817; doi:10.1016/j.ijid.2023.11.030)
Supplement: 7 [file NIHMS1958773-supplement-7.docx]

| **Table S2:** Subdistribution hazard ratios of progression from competing risk models among children with viral load measurements | | | | | | |
| --- | --- | --- | --- | --- | --- | --- |
|  |  |  |  |  |  |  |
| **Characteristics §** | | **Univariate** | **p value** |  | **Multivariable** | **p value** |
|  |  | **sHR (95% CI)** |  |  | **aHR_sd_ (95% CI)** |  |
| Country | |  | 0.852 |  |  | < .001 |
|  | Botswana | 1 |  |  | 1 |  |
|  | Uganda | 1.01 (0.87 – 1.18) |  |  | 0.40 (0.31 – 0.51) |  |
| Gender, *n (%)* | |  | 0.194 |  |  |  |
|  | Male | 1 |  |  |  |  |
|  | Female | 0.91 (0.80 – 1.05) |  |  |  |  |
| Age at Enrolment (per 1-yr increase) | |  |  |  |  |  |
|  |  | 0.11 (0.06 – 0.19) | < .001 |  | 0.04 (0.02 – 0.06) | < .001 |
| Year Birth | |  | < .001 |  |  |  |
|  | Before 2004 | 1 |  |  |  |  |
|  | After 2004 | 4.51 (3.25 – 6.26) |  |  |  |  |
| Year of enrolment | |  | 0.038 |  |  | < .001 |
|  | Before 2006 | 1 |  |  | 1 |  |
|  | 2006 – 2010 | 1.12 (0.91 – 1.38) |  |  | 0.36 (0.23 – 0.54) |  |
|  | After 2010 | 1.29 (1.05 – 1.59) |  |  | 2.10 (1.59 – 2.79) |  |
| HIV RNA load, (log10 copies/uL) | |  |  |  |  |  |
|  |  | 1.87 (1.52 – 2.30) | < .001 |  | 1.08 (0.76 – 1.53) | 0.001 |
| Weight-for-age z–score | |  | 0.004 |  |  |  |
|  | –1.75 | 1 |  |  |  |  |
|  | –3.75 | 1.06 (0.96 – 1.17) |  |  |  |  |
| Height-for-age z–score | |  | 0.022 |  |  |  |
|  | –1.75 | 1 |  |  |  |  |
|  | –-3.75 | 1.02 (0.89 – 1.17) |  |  |  |  |
| BMI-for-age z–score | |  | < .001 |  |  | 0.347 |
|  | –0.85 | 1 |  |  | 1 |  |
|  | –2.85 | 1.18 (1.06 – 1.30) |  |  | 1.17 (1.03 – 1.33) |  |
| § N = 847, † BMI-for-age was not included in any model with weight-for-age or height-for-age, BMI - body mass index | | | | | | |
|  |  |  |  |  |  |  |
